# Supplementary material for: Development of a Genome-Scale Metabolic Model of Clostridium thermocellum and Its Applications for Integration of Multi-Omics Datasets and Computational Strain Design
Source: Front Bioeng Biotechnol. 2020 Aug 21;8:772. doi: 10.3389/fbioe.2020.00772 (PMC7471609; doi:10.3389/fbioe.2020.00772)
Supplement: Supplementary Datasheet 3 — Supplementary tables. [file Data_Sheet_3.pdf]

Supplementary Material 3:
Development of a Genome-Scale Metabolic Model of *Clostridium thermocellum* and its Applications for Integration of Multi-Omics Datasets and Computational Strain Design

Sergio Garcia[1,2], R. Adam Thompson[2,3,6], Richard J. Giannone[2,5], Satyakam Dash[2,4], Costas D. Maranas[2,4], and Cong T. Trinh[1,2,3,*]

[1] *Department of Chemical and Biomolecular Engineering, The University of Tennessee, Knoxville, TN, USA.*
[2] *Center for Bioenergy Innovation, Oak Ridge National Laboratory, Oak Ridge, TN, USA.*
[3] *Bredesen Center for Interdisciplinary Research and Graduate Education, The University of Tennessee, Knoxville and Oak Ridge National Laboratory, Oak Ridge, TN, USA.*
[4] *Department of Chemical Engineering, The Pennsylvania State University, University Park, PA, USA.*
[5] *Chemical Sciences Division, Oak Ridge National Laboratory, Oak Ridge, TN, USA.*
[6] *Current affiliation: Quantitative Translational Pharmacology, DMPK-BA, Abbvie Inc., North Chicago, IL, USA.*
[*] *Corresponding author: 1512 Middle Dr, DO432, Department of Chemical and Biomolecular Engineering, University of Tennessee, Knoxville, TN 37996, USA. Tel: 865-974-2181. Email: ctrinh@utk.edu*

Table S1: A list of 70 consistent reactions in the $\Delta hydG\Delta ech$ case study and their associated fold changes. The biomass reaction is not included due to size. Note that this table is continued in the following pages.

ID	Formula	Fold change		
		proteomics	*pFBA*	*FVAcenter*
MDH	mal__L_c + nad_c ↔ h_c + nadh_c + oaa_c	1.4	0.2	0.1
PEPCK_re	co2_c + gdp_c + pep_c → gtp_c + oaa_c	0.9	0.1	0.1
VOR2b	3mob_c + coa_c + 2.0 fdxo_42_c → co2_c + 2.0 fdxr_42_c + h_c + ibcoa_c	0.9	12.3	0.8
PFL	coa_c + pyr_c → accoa_c + for_c	0.5	0.2	0.8
FRNDPR2r	2.0 fdxr_42_c + h_c + nadh_c + 2.0 nadp_c ↔ 2.0 fdxo_42_c + nad_c + 2.0 nadph_c	0.3	0.2	2.3
ALCD2x	acald_c + h_c + nadh_c → etoh_c + nad_c	0.1	0.8	0.6
IBUTCOARx	h_c + ibcoa_c + nadh_c → 2mppal_c + coa_c + nad_c	0.1	12.3	0.8
ACALD	accoa_c + h_c + nadh_c → acald_c + coa_c + nad_c	0.1	0.8	1.1
ALCD23xi	2mppal_c + h_c + nadh_c → ibutoh_c + nad_c	0.1	12.3	0.8
ME2	mal__L_c + nadp_c → co2_c + nadph_c + pyr_c	0.1	0.3	0.1
PSCVT	pep_c + skm5p_c → 3psme_c + pi_c	-0.1	-0.3	0.7
PTAr	accoa_c + pi_c ↔ actp_c + coa_c	-0.1	-2.7	-0.1
HSOR	3.0 h_c + 3.0 nadph_c + so3_c → 3.0 h2o_c + h2s_c + 3.0 nadp_c	-0.1	-0.3	-1.2
TRDR	h_c + nadph_c + trdox_c → nadp_c + trdrd_c	-0.1	-0.3	0.8
IGPS	2cpr5p_c + h_c → 3ig3p_c + co2_c + h2o_c	-0.1	-0.3	0.2
GLUDy	glu__L_c + h2o_c + nadp_c ↔ akg_c + h_c + nadph_c + nh4_c	-0.1	-0.5	-0.2
ECH	2.0 fdxr_42_c + 3.0 h_c ↔ 2.0 fdxo_42_c + h2_c + h_e	-0.1	-15.6	-13.5
ALLAS	24.0 ala__D_c + 24.0 atp_c + 24.0 cdpglyc_c + dg12dg_c + 24.0 h2o_c → ala_lta_c + 24.0 amp_c + 24.0 cmp_c + 48.0 h_c + 24.0 ppi_c	-0.1	-0.2	0.0
HSDxi	aspsa_c + h_c + nadh_c → hom__L_c + nad_c	-0.1	-0.3	0.8
PRMICI	prfp_c → prlp_c	-0.1	-0.3	-0.1
OCBT	cbp_c + orn__L_c ↔ citr__L_c + h_c + pi_c	-0.2	-0.3	-0.3
UAGCVT	pep_c + uacgam_c → pi_c + uaccg_c	-0.2	-0.3	-0.1
ANS	chor_c + gln__L_c → anth_c + glu__L_c + h_c + pyr_c	-0.2	-0.3	0.2
DHAD2	23dhmp_c → 3mop_c + h2o_c	-0.2	-0.3	0.6
ASPO2y	asp__L_c + nadp_c → h_c + iasp_c + nadph_c	-0.3	-0.3	-0.1
NADK	atp_c + nad_c → adp_c + h_c + nadp_c	-0.3	-0.3	-0.1
METS	5mthf_c + hcys__L_c → met__L_c + thf_c	-0.3	-0.3	0.8
IMPD	h2o_c + imp_c + nad_c ↔ h_c + nadh_c + xmp_c	-0.3	-0.3	-0.3
MG2abc	atp_c + h2o_c + mg2_e → adp_c + h_c + mg2_c + pi_c	-0.3	-0.3	-0.1
PDHam1hi	h_c + pyr_c + thmpp_c → 2ahethmpp_c + co2_c	-0.4	-0.3	12.4
ACAS_2ahbut	2ahethmpp_c + 2obut_c → 2ahbut_c + thmpp_c	-0.4	-0.3	0.6
GF6PTA	f6p_B_c + gln__L_c → gam6p_c + glu__L_c	-0.4	-0.3	0.0
CHRS	3psme_c → chor_c + pi_c	-0.4	-0.3	0.7
SERH	3ig3p_c + ser__L_c → g3p_c + h2o_c + trp__L_c	-0.4	-0.3	0.2
ALATA_L	akg_c + ala__L_c ↔ glu__L_c + pyr_c	-0.5	-0.3	-12.9
NNDPR	h_c + prpp_c + quln_c → co2_c + nicrnt_c + ppi_c	-0.5	-0.3	-0.1
TMDS	dump_c + mlthf_c → dhf_c + dtmp_c	-0.5	-0.3	-0.1
PHETA1	akg_c + phe__L_c ↔ glu__L_c + phpyr_c	-0.6	-0.3	0.8
TYRTA	akg_c + tyr__L_c ↔ 34hpp_c + glu__L_c	-0.6	-0.3	0.6
GMPS	atp_c + nh4_c + xmp_c → amp_c + gmp_c + 3.0 h_c + ppi_c	-0.6	-0.3	-0.3
SKK	atp_c + skm_c → adp_c + h_c + skm5p_c	-0.6	-0.3	0.7
ACOTA	acorn_c + akg_c ↔ acg5sa_c + glu__L_c	-0.6	-0.3	-0.3
PPDK	amp_c + 2.0 h_c + pep_c + ppi_c → atp_c + pi_c + pyr_c	-0.7	-10.0	0.1
GLUPRT	gln__L_c + h2o_c + prpp_c → glu__L_c + h_c + ppi_c + pram_c	-0.7	-0.3	-0.3
KARI	2ahbut_c ↔ cpd10162_c	-0.8	-0.3	0.6
KARI_23dhmp	23dhmp_c + nadp_c ↔ cpd10162_c + h_c + nadph_c	-0.8	-0.3	0.6
ARGSL	argsuc_c ↔ arg__L_c + fum_c	-0.8	-0.3	-0.3

ID	Formula	Fold change		
		proteomics	*pFBA*	*FVAcenter*
LEUTA	4mop_c + glu__L_c → akg_c + leu__L_c	-0.8	-0.3	0.4
ILETA	akg_c + ile__L_c ↔ 3mop_c + glu__L_c	-0.8	-0.3	0.6
VALTA	akg_c + val__L_c ↔ 3mob_c + glu__L_c	-0.8	-1.4	-1.5
ARGSS	asp__L_c + atp_c + citr__L_c ↔ amp_c + argsuc_c + 2.0 h_c + ppi_c	-0.8	-0.3	-0.3
SHSL2	h2s_c + suchms_c → hcys__L_c + succ_c	-0.9	-6.0	0.8
AHSL	achms_c + cys__L_c ↔ ac_c + cyst_L_c + h_c	-0.9	-10.6	-0.3
SHSL1	cyst_L_c + h_c + succ_c ↔ cys__L_c + suchms_c	-0.9	-10.6	0.4
ACKr	actp_c + adp_c → ac_c + atp_c	-0.9	-2.7	-0.1
QULNS	dhap_c + iasp_c → 2.0 h2o_c + h_c + pi_c + quln_c	-0.9	-0.3	-0.1
NADS2	atp_c + dnad_c + gln__L_c + h2o_c → amp_c + glu__L_c + 2.0 h_c + nad_c + ppi_c	-0.9	-0.3	-0.1
FE3abc	atp_c + fe3_e + h2o_c → adp_c + fe3_c + h_c + pi_c	-1.0	-0.3	-0.1
ASPTA	akg_c + asp__L_c ↔ glu__L_c + oaa_c	-1.0	-0.3	0.2
CTPS1	atp_c + nh4_c + utp_c → adp_c + ctp_c + 2.0 h_c + pi_c	-1.2	-0.3	0.0
ACGK	acglu_c + atp_c → acg5p_c + adp_c	-1.2	-0.3	-0.3
IGPDH	eig3p_c → h2o_c + imacp_c	-1.2	-0.3	-0.1
AGPR	acg5sa_c + nadp_c + pi_c ↔ acg5p_c + h_c + nadph_c	-1.3	-0.3	-0.3
PHEt2r	h_e + phe__L_e ↔ h_c + phe__L_c	-1.5	-0.3	0.8
UAG4Ei	uacgam_c → udpacgal_c	-1.5	-0.3	-0.1
CYSS	acser_c + h2s_c → ac_c + cys__L_c	-1.8	-0.3	-0.5
BIF	2.0 fdxr_42_c + 3.0 h_c + nadh_c ↔ 2.0 fdxo_42_c + 2.0 h2_c + nad_c	-1.8	-13.8	-12.5
UMPK	atp_c + h_c + ump_c → adp_c + udp_c	-2.1	-0.3	0.0
SULabc	atp_c + h2o_c + so4_e → adp_c + h_c + pi_c + so4_c	-4.6	-0.3	0.8

Table S2: The pFBA-simulated fluxes from the $\Delta hydG\Delta ech$ case study. In the table, we only presented reactions involving NADPH or exchange reactions that have different fluxes between the wild-type and mutant. We did not present the biomass reaction due to size as well as the reactions with the same fold change magnitude as the biomass reaction (|FC| = 0.26), likely because they are fully correlated.

ID	Formula	Fluxes (mmol/gCDW/hr)		
		W.T.	*Mut.*	FC
EX_ibutoh_e	ibutoh_e →	0.0	0.49	12.26
KARA1	alac__S_c + h_c + nadph_c → 23dhmb_c + nadp_c	0.22	0.59	1.42
EX_etoh_e	etoh_e →	1.09	1.88	0.78
EX_h2o_e	h2o_e ↔	-0.38	0.5	0.42
EX_co2_e	co2_e →	2.07	2.77	0.42
ME2	mal__L_c + nadp_c → co2_c + nadph_c + pyr_c	2.9	3.51	0.28
EX_for_e	for_e →	0.48	0.56	0.23
FRNDPR2r	2.0 fdxr_42_c + h_c + nadh_c + 2.0 nadp_c ↔ 2.0 fdxo_42_c + nad_c + 2.0 nadph_c	-1.02	-1.17	0.2
EX_nh4_e	nh4_e ↔	-0.73	-0.53	-0.48
GLUDy	glu__L_c + h2o_c + nadp_c ↔ akg_c + h_c + nadph_c + nh4_c	-0.61	-0.42	-0.53
EX_h_e	h_e ↔	2.93	1.19	-1.3
EX_val__L_e	val__L_e →	0.18	0.06	-1.54
ICDHyr	icit_c + nadp_c → akg_c + co2_c + nadph_c	0.21	0.04	-2.27
EX_ac_e	ac_e →	0.93	0.17	-2.46
EX_lac__L_e	lac__L_e →	0.05	0.01	-2.49
EX_succ_e	succ_e →	0.41	0.0	-12.01
EX_h2_e	h2_e →	2.2	0.0	-14.43
