# Supplementary material for: Development of a Genome-Scale Metabolic Model of Clostridium thermocellum and Its Applications for Integration of Multi-Omics Datasets and Computational Strain Design
Source: Front Bioeng Biotechnol. 2020 Aug 21;8:772. doi: 10.3389/fbioe.2020.00772 (PMC7471609; doi:10.3389/fbioe.2020.00772)
Supplement: Supplementary Datasheet 1 — Software used to develop, configure, and analyze iCBI655. [file Data_Sheet_1.ZIP › ctherm-gem-1.0.zip!/ctherm-gem-1.0/analysis/memote/iCBI655_cellobiose_batch.html]

MemoteReportApp
